# Single-Particle X‑ray Scattering Reveals a High Local Supersaturation of Precursors as the Origin of CoO Assembly Formation

**DOI:** 10.1021/acs.jpclett.6c00191

**Published:** 2026-06-01

**Authors:** Sani Y. Harouna-Mayer, Lars Klemeyer, Cecilia A. Zito, Johan Bielecki, Xuemei Cheng, Davide Derelli, Armando D. Estillore, Tjark L. R. Gröne, Lukas V. Haas, Romain Letrun, Chan Kim, Jayanath C. P. Koliyadu, Abhishek Mall, Parichita Mazumder, Diogo V. M. Melo, Adam R. Round, Amit K. Samanta, Abhisakh Sarma, Zhou Shen, Xiao Sun, Patrik Vagovic, Tamme Wollweber, Richard Bean, Jochen Küpper, Henry N. Chapman, Dorota Koziej, Kartik Ayyer

**Affiliations:** † Institute for Nanostructure and Solid-State Physics, Center for Hybrid Nanostructures, 530489 University of Hamburg, Hamburg 22761, Germany; ‡ European XFEL, 22869 Schenefeld, Germany; ¶ Center for Free-Electron Laser Science CFEL, Deutsches Elektronen-Synchrotron DESY, Hamburg 22607, Germany; § 14915 Max Planck Institute for the Structure and Dynamics of Matter, Hamburg 22761, Germany; ∥ Deutsches Elektronen-Synchrotron DESY, Hamburg 22607, Germany; ⊥ Department of Physics, Universität Hamburg, 22761 Hamburg, Germany; # The Hamburg Center for Ultrafast Imaging, Hamburg 22761, Germany; ○ Institute of Integrated Natural Science, University of Koblenz, Koblenz 56070, Germany

## Abstract

Single-particle small-angle X-ray scattering (SP-SAXS) at X-ray free electron lasers (XFELs) enables quantitative analysis of morphological heterogeneity that is fundamentally inaccessible to ensemble-averaged *in situ* techniques. By recording diffraction snapshots from isolated particles, SP-SAXS resolves low-contrast, less abundant, or transient species within heterogeneous particle populations that would otherwise remain hidden to conventional X-ray techniques. We demonstrate this unique capability by investigating the solvothermal formation of CoO nanocrystal assemblies from a Co­(acac)_3_ precursor in benzyl alcohol. The single-particle data revealed amorphous, uniform-density Co­(acac)_2_ spheres as transient intermediates that directly crystallize into cavernous CoO nanocrystal assemblies, explaining why CoO forms as hierarchical aggregates rather than as isolated nanocrystals. These results establish SP-SAXS as a uniquely powerful framework for uncovering nonclassical nanoparticle formation pathways hidden in ensemble measurements.

## Graphical abstract

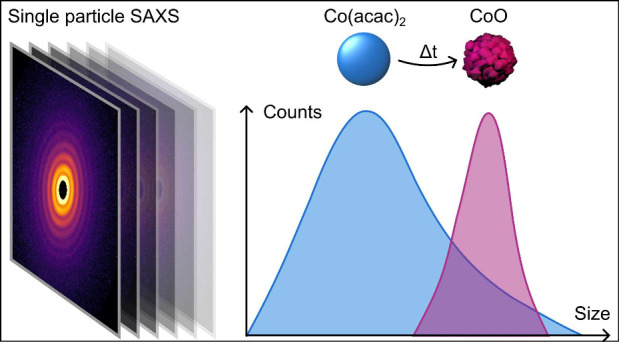

The emergence of nanomaterials in solution is governed by complex chemical and structural transformations that ultimately dictate their composition, structure, morphology, and functionality. The rational design of nanomaterials with tailored properties therefore requires mechanistic insight into their formation pathways.^1–3^ In many systems, nanomaterials do not form through the straightforward monomer-by-monomer growth described by classical nucleation theory but rather follow nonclassical pathways involving metastable intermediates such as prenucleation clusters, dense liquid phases, amorphous precipitates, or the assembly of nanoscale building blocks into hierarchical architectures.^4–6^ These multistep routes have been reported across a wide range of material classes, yet they continue to pose significant challenges for mechanistic understanding and predictive control.^7–11^

Among the most powerful methods for investigating nanomaterial formation are X-ray techniques at synchrotron sources.^12^ For instance, wide-angle X-ray scattering (WAXS) provides access to atomic arrangements, while small-angle X-ray scattering (SAXS) probes particle size, shape, and morphology.^13,14^ X-ray absorption spectroscopy (XAS) offers element-specific insight into the electronic structure and chemical environment of the absorbing atom.^15^ Complementary optical spectroscopies such as ultraviolet, visible, and infrared (UV/vis/IR) spectroscopy are sensitive to organic species, optical band gap transitions, and plasmonic resonances.^16^ Similar to SAXS, dynamic light scattering (DLS) probes the particle size, but it assumes a hard sphere model and is not applicable to broad or multimodal size distributions.^17^ Such methods provide comprehensive information about nanoparticle formation and can be applied *in situ*, enabling real-time monitoring of the evolution of the electronic, atomic, and mesoscopic structure. However, they inherently average over the illuminated sample volume, which may obscure structural or chemical heterogeneity within particle ensembles.^18^ The analytical ultracentrifugation (AUC) enables the deconvolution of particle size distributions from sedimentation profiles of colloidal nanoparticle dispersions. However, it relies on assumptions about particle density, shape, and frictional ratio, and thus cannot accurately resolve heterogeneous, complex, or anisotropic morphologies.^19^

In contrast, individual particles can be directly imaged during formation in solution using *in situ* electron microscopy (EM) or from quenched aliquots via cryogenic (cryo-)­EM, which, however, require elaborate sample preparation, and are prone to electron-beam-induced damage and confinement effects, and only very small sample volumes can be probed.^20^ Similarly, atomic force microscopy (AFM) can resolve surface morphology and size distributions of deposited nanoparticles but is limited to dried samples and small surface areas. In summary, all conventional methods which allow the study of nanomaterial formation mechanisms either lose information by averaging over the whole sample volume, or only allow very small sample quantities and might be further altered due to sample preparation or beam-damage.

Here, we introduce single-particle small-angle X-ray scattering (SP-SAXS), which enables the morphological analysis of very large numbers of individual particles using an X-ray free-electron laser (XFEL). The ultrashort and extremely intense XFEL pulses used in SP-SAXS ensure that diffraction is recorded before the onset of X-ray-induced damage, effectively capturing an undistorted structural snapshot of each particle.^21^ A related technique, termed X-ray single-particle imaging (SPI), was first developed to determine the structure of biomolecules without crystallization^22–24^ and has been applied to study heterogeneous ensembles in the context of imaging their morphological variations.^25–27^

We apply SP-SAXS to find transient intermediates in a model chemical synthesis, the solvothermal formation of CoO nanocrystal assemblies from a Co­(acac)_3_ precursor in benzyl alcohol at 160 °C. Previous complementary *in situ* X-ray studies followed the reaction from the molecular precursor to the final assemblies by combining XAS with WAXS and SAXS, providing a comprehensive and internally consistent picture of the chemical reduction, nucleation, and growth steps.^28^ In particular, XAS revealed the rapid reduction of Co^3+^ to Co^2+^ and identified Co­(acac)_2_ as a stable intermediate, which gradually transformed into rock-salt CoO. Time-resolved WAXS and SAXS analyses showed that both CoO nanocrystals and corresponding spherical assemblies grew concurrently over the course of the reaction.

However, by their very nature, ensemble-averaged *in situ* techniques probe the average response of all species present in the reaction volume. As a result, distinct formation scenarios, such as aggregation of nanocrystals versus crystallization within amorphous precursor entities, could not be disentangled. Consequently, even this thorough multimodal characterization could not, in principle, resolve the morphological identity of transient intermediates or establish the structural origin of CoO assembly formation.

By analyzing scattering patterns from individual CoO assemblies and preassembly entities extracted from the reaction solution during the early stages of assembly formation, we identify amorphous uniform-density spheres as transient intermediates, that subsequently crystallize into cavernous superstructures. This single-particle perspective provides the missing mechanistic link and explains why CoO forms as assemblies rather than as dispersed nanocrystals. Importantly, the present findings are fully consistent with earlier *in situ* observations, while extending them by accessing a complementary level of structural information that is fundamentally absent from ensemble-averaged measurements.

Figure 1 illustrates the experimental and analytical workflow of conventional SAXS in comparison with SP-SAXS. In conventional SAXS, measured at a synchrotron or laboratory X-ray source, each diffraction pattern represents the sum of scattering contributions from all species within the illuminated sample volume. In contrast, SP-SAXS collects diffraction patterns from individual particles that are delivered in a dilute aerosol or liquid jet at an XFEL. The single-particle diffraction patterns are typically noisy, incomplete, and unoriented. To obtain high-resolution data, a large ensemble of similar single-particle diffraction patterns are identified, orientationally aligned, and averaged. Each averaged data set forms a class, whose relative hit ratio reflects the population of the corresponding particle type within the sample. The SP-SAXS data processing routine follows similar principles to single-particle imaging (SPI) or coherent diffractive imaging (CDI), in which the individual diffraction patterns are mapped in three-dimensional diffraction space and phase reconstructed.^25,29^ In SP-SAXS, we analyze the averaged two-dimensional diffraction images and their radial integrations.

**Figure 1:**
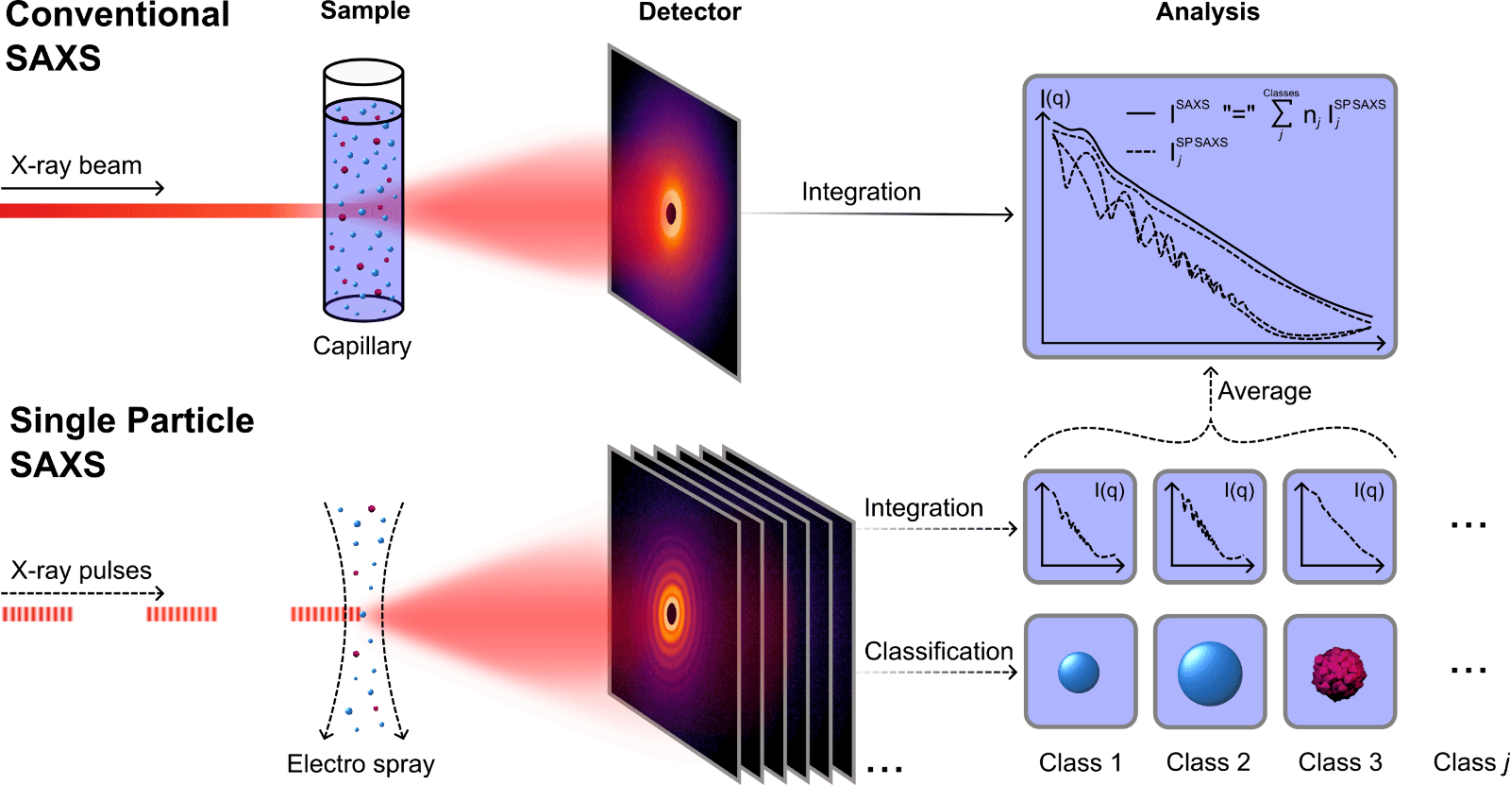
Experimental and analytical workflow of single-particle SAXS (SP-SAXS) compared to conventional SAXS. In conventional SAXS, the diffraction pattern comprises scattering contributions of all species within the illuminated sample volume of the X-ray beam from a synchrotron or laboratory source. In SP-SAXS, diffraction patterns from individual particles are averaged into classes, each representing a distinct particle population within the sample. The relative hit ratio of each class, n_*j*_, reflects the concentration of the corresponding particle species *j*. In principle, the sum of all SP-SAXS class diffraction patterns, I_*j*_^SP‑SAXS^, reproduces the total diffraction pattern obtained in conventional SAXS, I^SAXS^.

To elucidate the CoO nanocrystal assembly formation pathway, we perform SP-SAXS on reaction aliquots collected at three early reaction times during the emergence of the CoO assemblies: 20, 30, and 40 min. In total, we collect 650 000 single-particle diffraction snapshots with an average hit rate of 2.1%, from which 60 distinct classes are identified across the combined data set of the three aliquots. Table S1 lists all classes including their total hit rate and relative occupancies across the different reaction times. Figures S1 and S2 display the diffraction images and corresponding radial integrations of all classes.

Although the focused XFEL beam, with a diameter of approximately 250–300 nm, is larger than the particles, most X-ray pulses interact with no particle, while a small fraction produce a measurable single-particle scattering pattern. Since individual single-shot patterns contain only a few photons at high *q*, structurally similar patterns are classified and averaged for further analysis. In Figure 2, we show representative aligned average diffraction patterns of selected classes.

**Figure 2:**
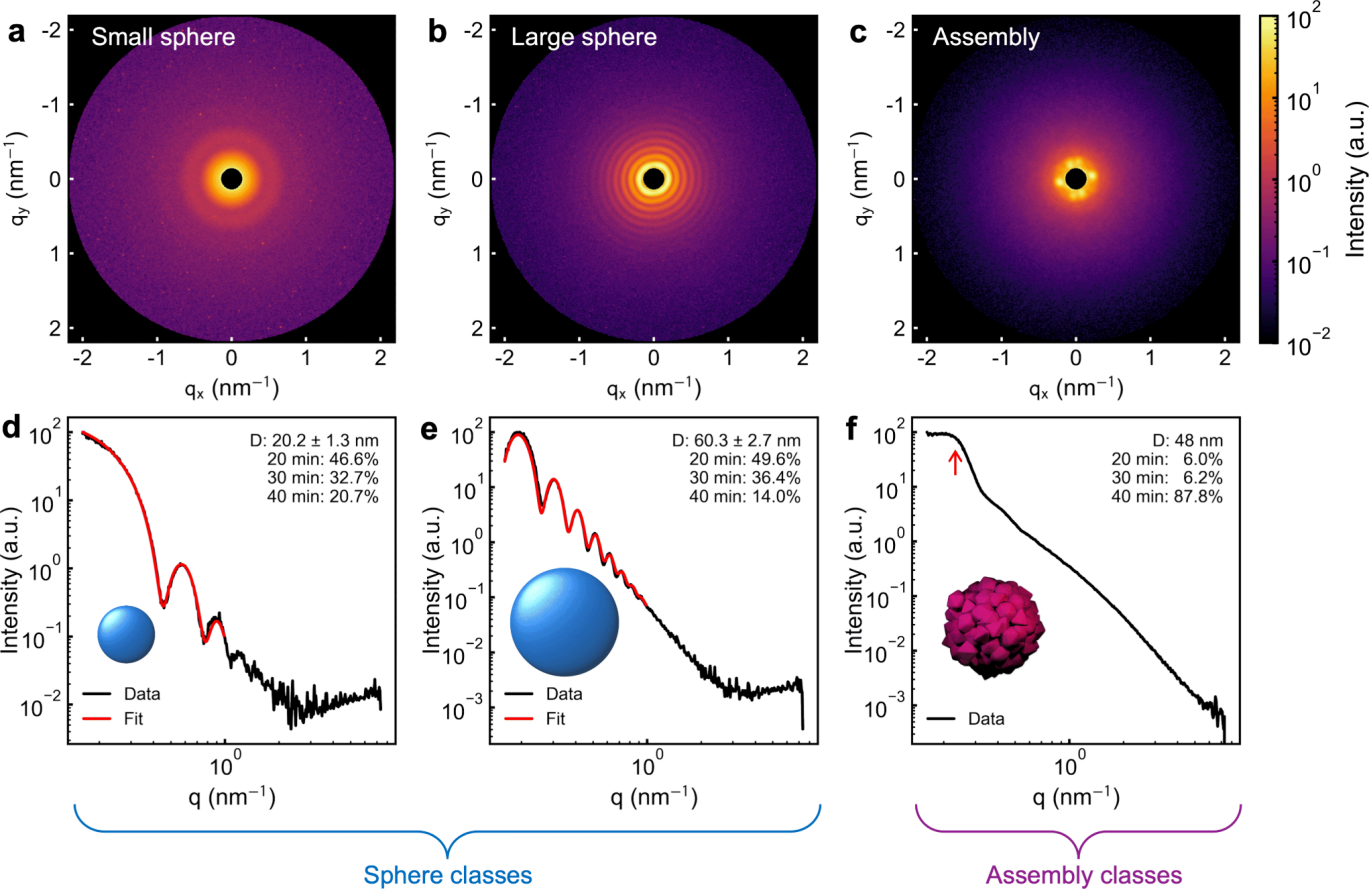
Representative diffraction images (a–c) and corresponding radial integrations (d–f) of selected SP-SAXS classes. The diameter D of the sphere classes is fitted with a spherical form factor, where the error represents the standard deviation of the Gaussian distribution. The diameter of the assembly classes is estimated by the intensity bump maximum as marked by the red arrow. The relative occupancy of each class at 20, 30, and 40 min reflects the temporal evolution of the populations.

The scattering profiles can be assigned either to amorphous, uniform-density spheres, referred to as sphere classes, or to nanocrystal assemblies, referred to as assembly classes. The sphere classes exhibit isotropic ring patterns in their diffraction images, and the corresponding radial integrations display the characteristic oscillations of monodisperse spherical form factors with an overall q^–4^ intensity decay. At higher q values >3 nm^–1^, the intensity increases systematically in all sphere classes, which originates from diffuse scattering from the amorphous structure of the spherical particles. The assembly classes, in contrast, display sharp low-q peaks in the diffraction images, arising from the internal fractal arrangement of nanocrystals within the assemblies. Their radial integrations typically feature one intensity bump around 0.2 nm^–1^, followed by a smooth decay, closely resembling the SAXS profile observed after full conversion of the intermediate into CoO assemblies.^28^ We estimate the assembly size from the position of the intensity bump. The sphere patterns are modeled using a spherical form factor, incorporating a Gaussian size distribution to account for minor variations in particle size within each sphere class. We note that some sphere classes fit well in the low-q region of the first fringes, but the model tends to underestimate the intensity for q > 0.5 nm^–1^. This weak deviation suggests the onset of structural inhomogeneity, possibly early crystallization within a subset of spheres; however, the effect is subtle and should be regarded as a qualitative trend rather than a quantitative indicator of structural evolution. Synthesis, sample preparation, SP-SAXS data processing, and the fitting procedure and size determination of the classes’ diffraction patterns are described in the Supporting Information in detail.

Figure 3a–c shows the summed radial integrations of the sphere, assembly, and all classes at the different reaction times. The summed sphere classes show a steady q^–4^ slope, due to smearing of spherical form factor oscillations of the overall polydisperse ensemble, and a positive slope at high q due to the diffuse scattering of the amorphous spheres. The summed assembly classes radial integrations exhibit the characteristic low q intensity bump associated with the internal nanocrystal arrangement within the assemblies. At reaction times 20 and 30 min, the scattering contribution from the assembly classes is indistinguishable in the radial integration sum of all classes whereas at 40 min the assembly classes dominate the scattering profile due to their increasing concentration. Figure 3d–f shows the size distributions of the sphere and assembly classes at the respective reaction times, revealing a progressive increase in the fraction of assemblies over time.

**Figure 3:**
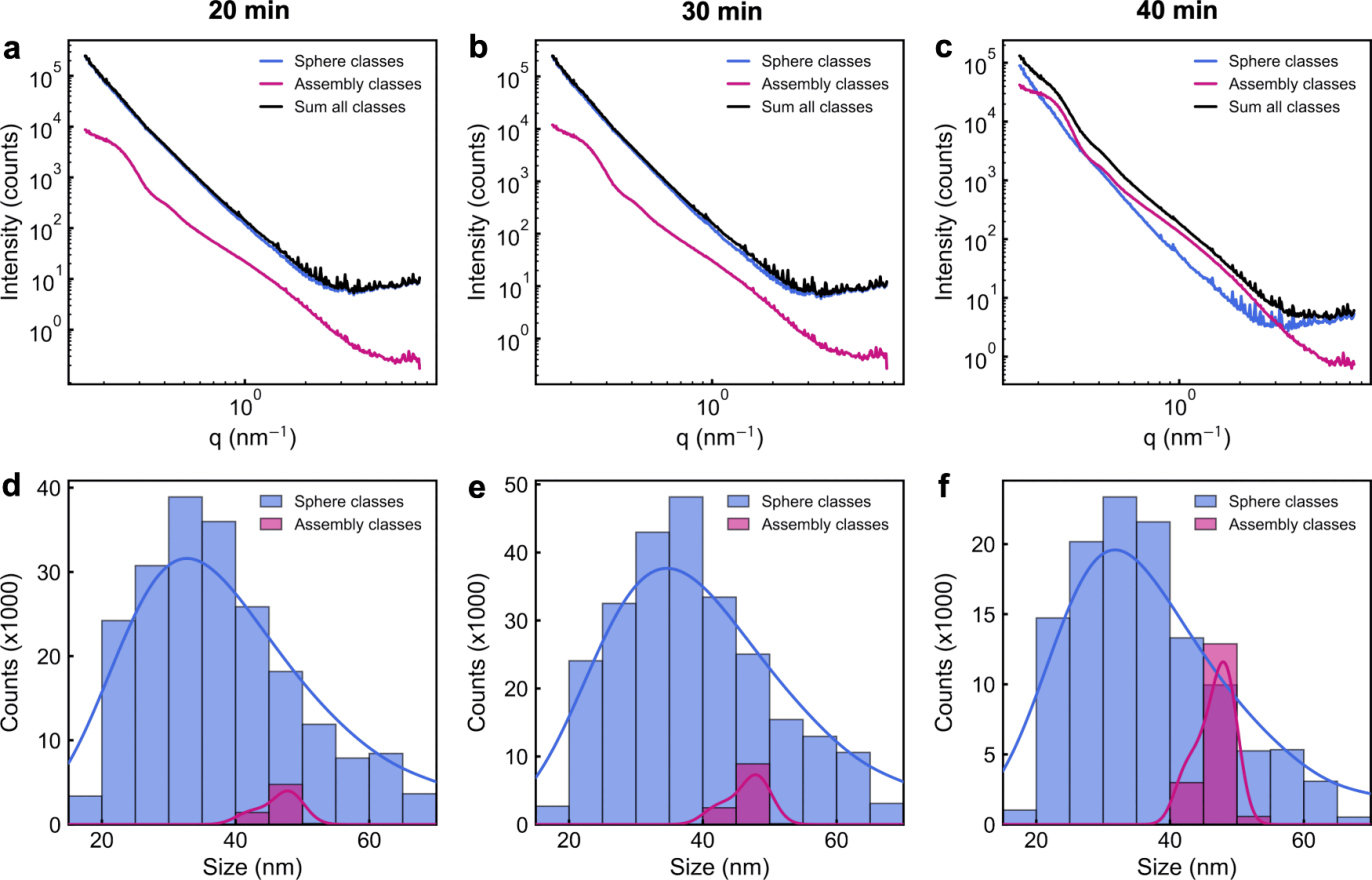
SP-SAXS analysis of the reaction aliquots at 20, 30, and 40 min. (a–c) Summed radial integrations of the sphere, assembly, and all classes. (d–f) Histograms of the size distribution of the sphere and assembly classes. The solid trace shows a kernel density estimate of the histograms.

Altogether, the SP-SAXS analysis reveals a population evolution from amorphous, uniform-density spheres to nanocrystal assemblies, as illustrated in Figure 4. To interpret these morphological observations, we relate the SP-SAXS results to the chemical transformation pathway established in earlier *in situ* X-ray studies: Initially, the precursor Co­(acac)_3_ is dissolved in benzyl alcohol, where it reduces to the intermediate Co­(acac)_2_, which subsequently transforms into CoO.^28^ The SP-SAXS findings indicate that Co­(acac)_2_ phase-separates into spherical amorphous precipitates upon reduction, owing to its low solubility in benzyl alcohol. The comparable size range of these amorphous spheres and the emerging assemblies suggests a direct structural transformation rather than secondary aggregation of individual nanocrystals. Crystallization is likely initiated from high local supersaturation of Co­(acac)_2_ inside the precipitate volume. During crystallization, the higher density of CoO compared to Co­(acac)_2_ causes the spherical precipitates to contract, giving rise to cavernous polycrystalline assemblies instead of dense crystalline entities.

**Figure 4:**
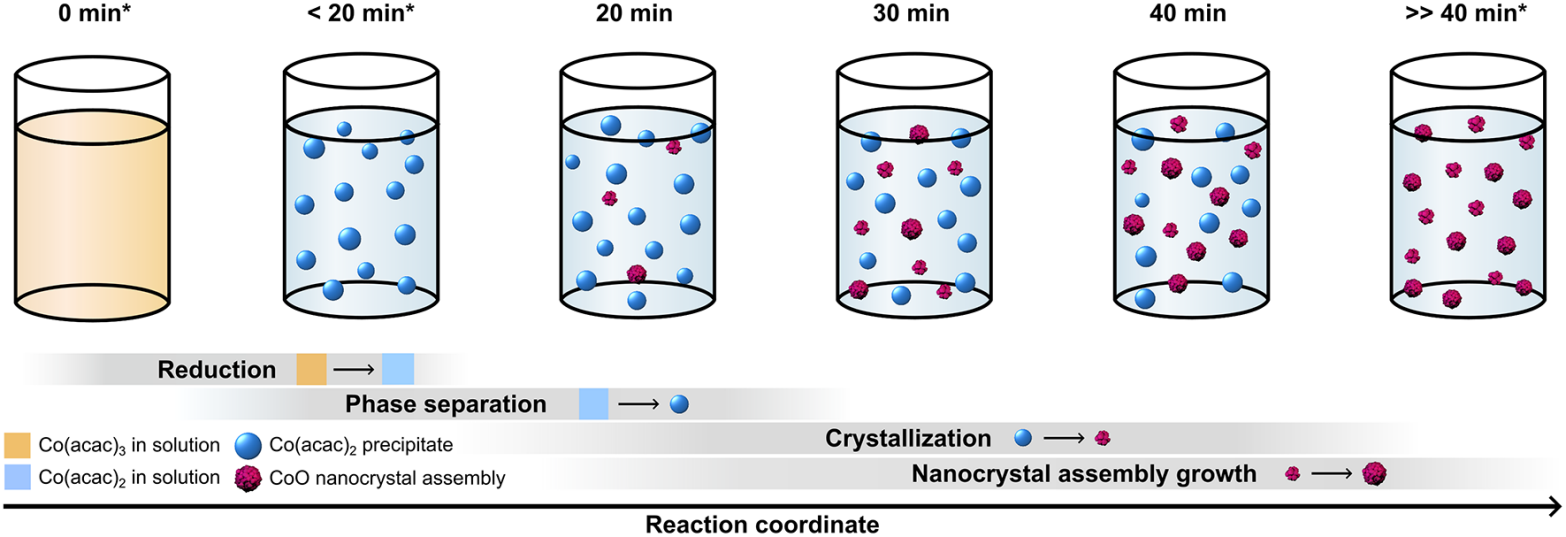
Schematic illustration of the proposed formation pathway of CoO nanocrystal assemblies after the reaction of Co­(acac)_3_ in benzyl alcohol. Initially, Co­(acac)_3_ reduces to Co­(acac)_2_, which subsequently phase-separates to spherical amorphous precipitates. With increasing reaction time, these Co­(acac)_2_ precipitates crystallize into CoO nanocrystal assemblies. The asterisks (*) denote extrapolated reaction states before and after the measured time points of 20, 30, and 40 min.

The Co­(acac)_2_ spheres can easily be overlooked in conventional SAXS measurements, as their smooth intensity decay lacks distinct features in the SAXS regime and may be mistaken for background scattering. In electron microscopy (EM) images, these spheres can also be misinterpreted as organic aggregates or reaction byproducts unrelated to the assembly formation mechanism. Moreover, because of the poor solubility of Co­(acac)_2_ in benzyl alcohol, a significant fraction of the spheres may be lost during sample washing and redispersion. For instance, spherical aggregates are found in the supernatant after washing the reaction mixture with ethanol, as shown in Figure S7a,b. This likely explains why the spherical Co­(acac)_2_ particles were not observed in previous studies, where the samples were washed several times with ethanol prior to EM analysis.^28^ To further confirm the precipitation behavior of Co­(acac)_2_, Figure S7c,d shows EM images of commercial Co­(acac)_2_ dissolved in benzyl alcohol and ethanol, both showing precipitation of spherical particles similar to those detected in the reaction solution and supernatant.

In conclusion, the single-particle perspective provided by SP-SAXS resolves a mechanistic question inaccessible to even the most comprehensive ensemble-averaged *in situ* characterization^28^ by quantitatively resolving distinct particle populations that were obscured in conventional measurements. SP-SAXS revealed that intermediate amorphous Co­(acac)_2_ spheres crystallize into CoO nanocrystal assemblies, elucidating why CoO emerges as hierarchical aggregates rather than as dispersed nanocrystals. Beyond this specific case, SP-SAXS represents a broadly applicable approach for studying complex reaction and formation pathways in complex systems. Extending the concept to single-particle wide-angle X-ray scattering (SP-WAXS), achieved by reducing the sample-to-detector distance, would allow quantitative access to atomic-scale order similar to serial femtosecond crystallography (SFX).^30^ A multimodal two-detector configuration could further combine SP-SAXS and SP-WAXS, bridging the full range from atomic to mesoscopic structure. Looking ahead, the realization of *in situ* SP-SAXS and SP-WAXS experiments, where small volumes of the reaction mixture are continuously injected into the XFEL beam, will open the way toward real-time visualization of nanoparticle nucleation and growth at the single-particle level, transforming our ability to directly observe matter in formation.

Although the reaction studied here evolves on minute time scales, the XFEL enables single-particle analysis from rare intermediates, avoiding averaging of details due to heterogeneity. The femtosecond pulse duration freezes the particle structure before radiation damage develops, while the high peak coherent flux provides sufficient signal from one particle at a time. The present experiment therefore illustrates a complementary use of XFELs in materials chemistry, not only for femtosecond dynamics but also for measuring heterogeneous structural distributions in relatively slow, nonequilibrium synthesis processes.

## Supplementary Material





## Notes

Diffraction images of all 60 SP-SAXS classes; radial integrations of all SP-SAXS classes with diameters and relative occupancies; table of all SP-SAXS classes with classification, particle sizes, relative occupancies, number of hits, and hit ratios; experimental details and data-classification procedure of the SP-SAXS measurements; size-determination procedures for the sphere and assembly classes; synthesis procedure for CoO nanocrystal assemblies; sample-preparation protocols for SP-SAXS, conventional SAXS, pair distribution function, and electron-microscopy measurements; conventional SAXS data of reaction solutions in benzyl alcohol and ethanol; experimental and simulated pair distribution functions of the 20, 30, and 40 min samples; transmission electron microscopy images of CoO nanocrystal assemblies ; scanning transmission electron microscopy images of spherical aggregates from solutions in benzyl alcohol and ethanol; discussion of the apparent shift of the scattering minimum and its correlation with the fitted size-distribution width; discussion of the synthesis reproducibility and SEM image of rose-like particles; and experimental details of the electron microscopy, conventional SAXS, and total X-ray scattering/pair distribution function analysis methods (PDF)
Transparent Peer Review report available (PDF)
