# Supplementary material for: Single-Particle X‑ray Scattering Reveals a High Local Supersaturation of Precursors as the Origin of CoO Assembly Formation
Source: J Phys Chem Lett. 2026 Jun 1;17(23):6418–24. doi: 10.1021/acs.jpclett.6c00191 (PMC13267086; doi:10.1021/acs.jpclett.6c00191)
Supplement: Supplementary file 1 [file jz6c00191_si_001.pdf]

Supporting Information for:

Single-Particle X-ray Scattering Reveals a High Local Supersaturation of Precursors as the Origin of CoO Assembly Formation

Sani Y. Harouna-Mayer,[†,#] Lars Klemeyer,[†,#] Cecilia A. Zito,[†,#] Johan Bielecki,[‡] Xuemei Cheng,[¶] Davide Derelli,[†] Armando D. Estillore,[¶] Tjark L. R. Groene,[†] Lukas V. Haas,[¶,#] Romain Letrun,[‡] Chan Kim,[‡] Jayanath C. P. Koliyadu,[‡] Abhishek Mall,[§] Parichita Mazumder,[§,#] Diogo V. M. Melo,[‡] Adam R. Round,[‡] Amit K. Samanta,[¶,#] Abhisakh Sarma,[‡] Zhou Shen,[§] Xiao Sun,[‖,@] Patrik Vagovic,[‡] Tamme Wollweber,[§,#] Richard Bean,[‡] Jochen Küpper,[¶] Henry N. Chapman,[¶,⊥,#] Dorota Koziej,[*,†,#] and Kartik Ayyer[*,§,#]

†*Institute for Nanostructure and Solid-State Physics, Center for Hybrid Nanostructures, University of Hamburg, Hamburg 22761, Germany*

‡*European XFEL, 22869 Schenefeld, Germany*

¶*Center for Free-Electron Laser Science CFEL, Deutsches Elektronen-Synchrotron DESY, Hamburg 22607, Germany*

§*Max Planck Institute for the Structure and Dynamics of Matter, Hamburg 22761, Germany*

‖*Deutsches Elektronen-Synchrotron DESY, Hamburg 22607, Germany*

⊥*Department of Physics, Universität Hamburg, 22761 Hamburg, Germany*

#*The Hamburg Center for Ultrafast Imaging, Hamburg 22761, Germany*

@*Institute of Integrated Natural Science, University of Koblenz, Koblenz 56070, Germany*

E-mail: dorota.koziej@uni-hamburg.de; kartik.ayyer@mpsd.mpg.de

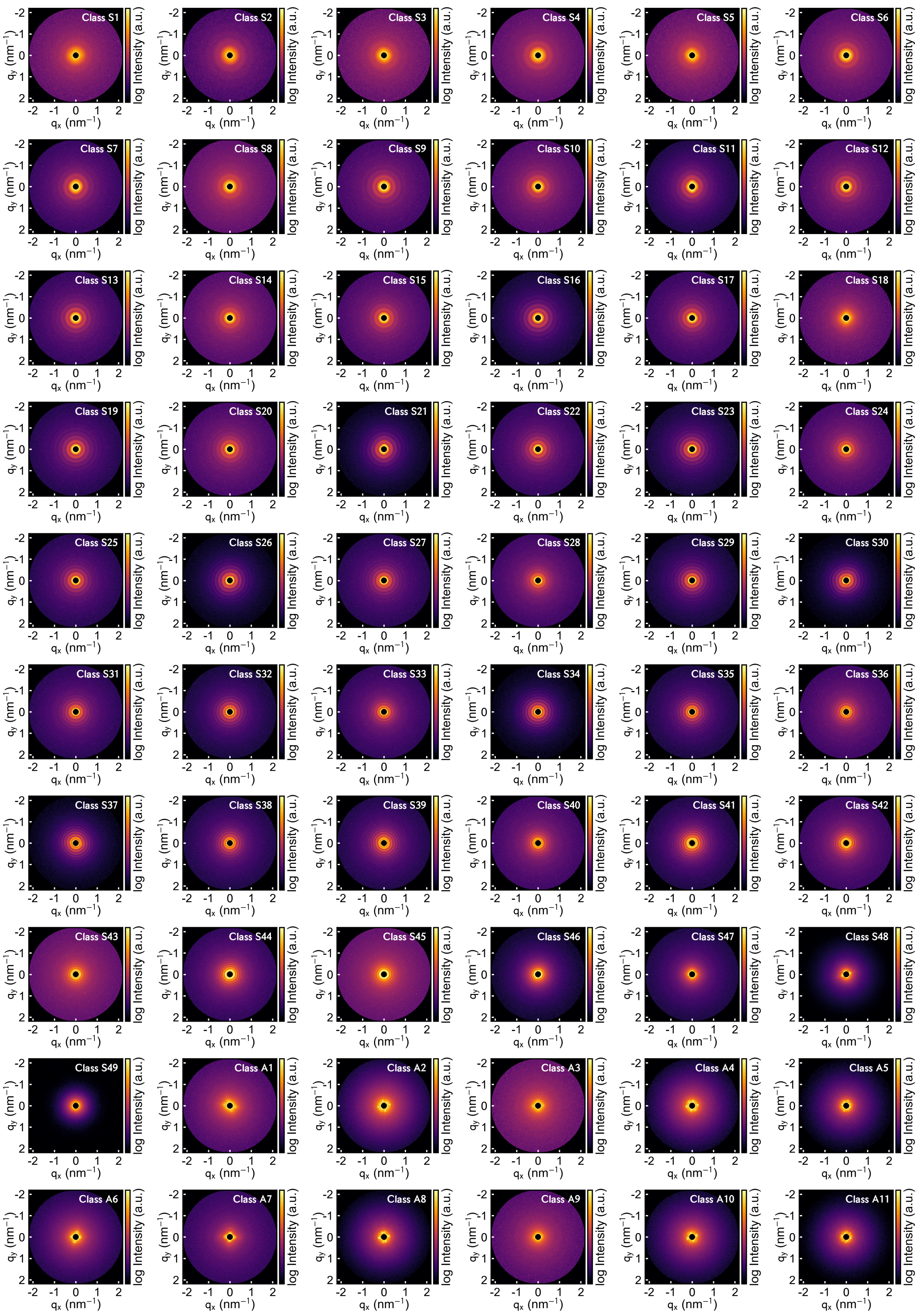

Figure S1: Diffraction images of all SP-SAXS classes.

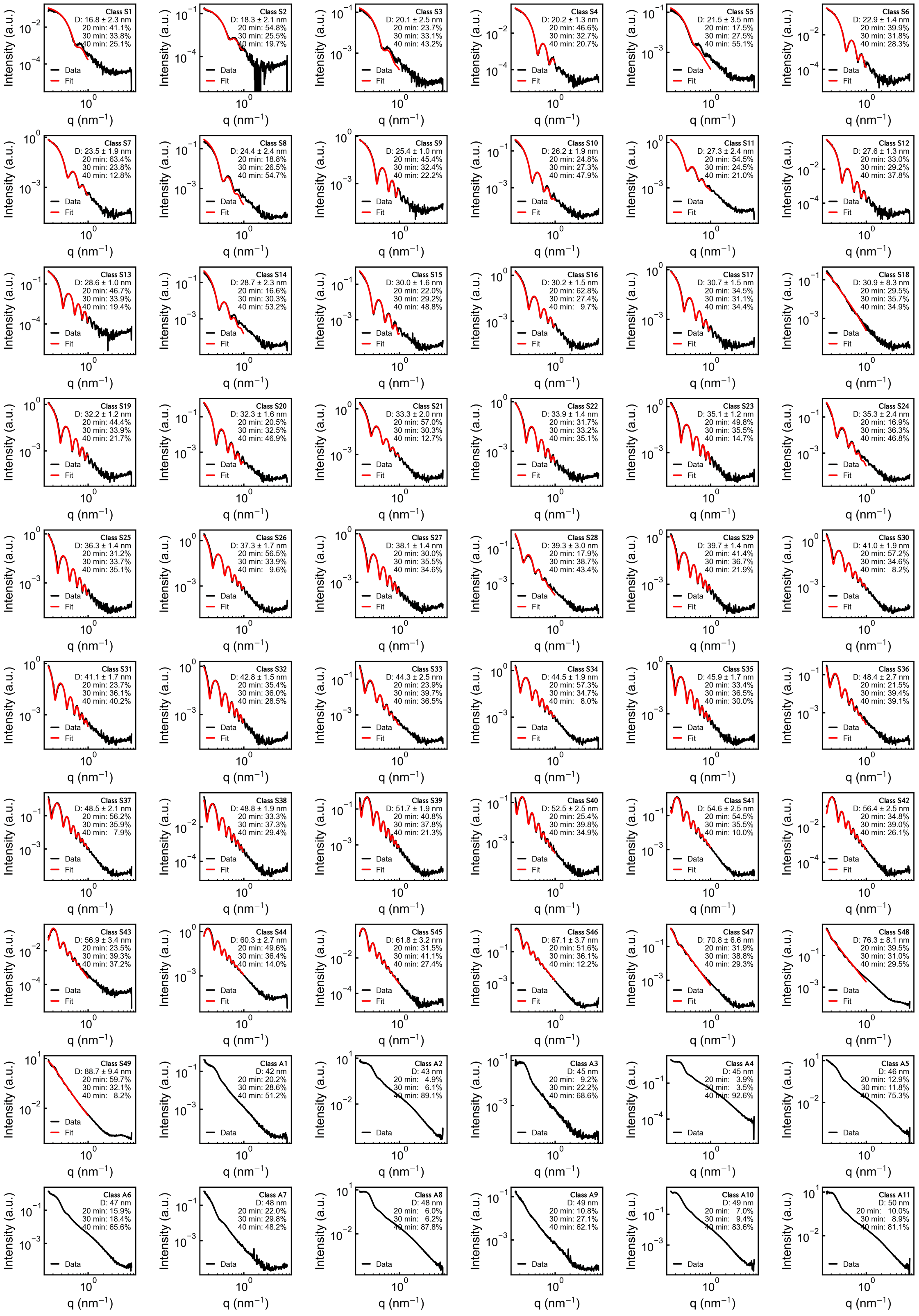

Figure S2: Radial integration of all SP-SAXS classes including their determined diameter D and relative occupancy of the measured reaction times 20, 30, and 40 min.

Table S1: List of all SP-SAXS classes with class number (#), their classification into sphere (S) or assembly (A) classes, determined particle sizes, relative occupancies at the different reaction times (20, 30, and 40 min), total number of hits per class, and the hit ratio of each class relative to the total number of hits across all classes.

#	Diameter (nm)	20 min (%)	30 min (%)	40 min (%)	No. hits	Hit ratio (%)
S1	16.8 ± 2.3	41.1	33.8	25.1	4333	0.0067
S2	18.3 ± 2.1	54.8	25.5	19.7	2771	0.0043
S3	20.1 ± 2.5	23.7	33.1	43.2	3280	0.0051
S4	20.2 ± 1.3	46.6	32.7	20.7	15237	0.0237
S5	21.5 ± 3.5	17.5	27.5	55.1	3090	0.0048
S6	22.9 ± 1.4	39.9	31.8	28.3	14541	0.0226
S7	23.5 ± 1.9	63.4	23.8	12.8	8776	0.0137
S8	24.4 ± 2.4	18.8	26.5	54.7	18100	0.0282
S9	25.4 ± 1.0	45.4	32.4	22.2	13540	0.0211
S10	26.2 ± 1.9	24.8	27.3	47.9	16331	0.0254
S11	27.3 ± 2.4	54.5	24.5	21.0	9984	0.0155
S12	27.6 ± 1.3	33.0	29.2	37.8	14975	0.0233
S13	28.6 ± 1.0	46.7	33.9	19.4	12581	0.0196
S14	28.7 ± 2.3	16.6	30.3	53.2	16025	0.0249
S15	30.0 ± 1.6	22.0	29.2	48.8	16310	0.0254
S16	30.2 ± 1.5	62.8	27.4	9.7	9894	0.0154
S17	30.7 ± 1.5	34.5	31.1	34.4	16396	0.0255
S18	30.9 ± 8.3	29.5	35.7	34.9	4160	0.0065
S19	32.2 ± 1.2	44.4	33.9	21.7	13113	0.0204
S20	32.3 ± 1.6	20.5	32.5	46.9	17880	0.0278
S21	33.3 ± 2.0	57.0	30.3	12.7	9569	0.0149
S22	33.9 ± 1.4	31.7	33.2	35.1	17933	0.0279
S23	35.1 ± 1.2	49.8	35.5	14.7	12412	0.0193
S24	35.3 ± 2.4	16.9	36.3	46.8	16678	0.0260
S25	36.3 ± 1.4	31.2	33.7	35.1	17124	0.0267
S26	37.3 ± 1.7	56.5	33.9	9.6	12365	0.0192
S27	38.1 ± 1.4	30.0	35.5	34.6	16589	0.0258
S28	39.3 ± 3.0	17.9	38.7	43.4	16164	0.0252
S29	39.7 ± 1.4	41.4	36.7	21.9	14393	0.0224
S30	41.0 ± 1.9	57.2	34.6	8.2	9600	0.0149
S31	41.1 ± 1.7	23.7	36.1	40.2	18259	0.0284
S32	42.8 ± 1.5	35.4	36.0	28.5	15901	0.0248
S33	44.3 ± 2.5	23.9	39.7	36.5	19276	0.0300
S34	44.5 ± 1.9	57.3	34.7	8.0	9581	0.0149
S35	45.9 ± 1.7	33.4	36.5	30.0	15587	0.0243
S36	48.4 ± 2.7	21.5	39.4	39.1	15322	0.0238
S37	48.5 ± 2.1	56.2	35.9	7.9	8394	0.0131
S38	48.8 ± 1.9	33.3	37.3	29.4	13871	0.0216
S39	51.7 ± 1.9	40.8	37.8	21.3	10418	0.0162
S40	52.5 ± 2.5	25.4	39.8	34.9	15325	0.0239
S41	54.6 ± 2.5	54.5	35.5	10.0	6836	0.0106
S42	56.4 ± 2.5	34.8	39.0	26.1	13104	0.0204
S43	56.9 ± 3.4	23.5	39.3	37.2	13076	0.0204
S44	60.3 ± 2.7	49.6	36.4	14.0	8262	0.0129
S45	61.8 ± 3.2	31.5	41.1	27.4	13832	0.0215
S46	67.1 ± 3.7	51.6	36.1	12.2	7288	0.0113
S47	70.8 ± 6.6	31.9	38.8	29.3	17887	0.0278
S48	76.3 ± 8.1	39.5	31.0	29.5	10107	0.0157
S49	88.7 ± 9.4	59.7	32.1	8.2	1777	0.0028
A1	42	20.2	28.6	51.2	5813	0.0090
A2	43	4.9	6.1	89.1	1054	0.0016
A3	45	9.2	22.2	68.6	3459	0.0054
A4	45	3.9	3.5	92.6	814	0.0013
A5	46	12.9	11.8	75.3	1103	0.0017
A6	47	15.9	18.4	65.6	3581	0.0056
A7	48	22.0	29.8	48.2	9831	0.0153
A8	48	6.0	6.2	87.8	938	0.0015
A9	49	10.8	27.1	62.1	4950	0.0077
A10	49	7.0	9.4	83.6	1876	0.0029
A11	50	10.0	8.9	81.1	798	0.0012
$\sum$	-	35.3	42.9	21.8	642461	100.0000

Experimental

Single particle small-angle X-ray scattering (SP-SAXS):

The SP-SAXS measurements were performed at the Single Particle, Biomolecules and Clusters/Serial Femtosecond Crystallography (SPB/SFX) end-station at the European X-ray Free Electron Laser (EuXFEL).[1] X-ray pulses with photon energy of 6 keV and average pulse energy of 1.2 µJ were focused to a diameter of around 250 nm. The sample dispersion was aerosolized and transported to the X-ray interaction region using an electrospray and aerodynamic lens stack injection system.[2] Diffraction patterns were collected at an average rate of 3420 frames/second in 10 bursts of 342 frames per second on the AGIPD-1M detector[3] placed 700 mm downstream of the interaction point.

An average of 2.0 % of the patterns contained statistically significant diffraction from single particles above the background scattering, primarily from the carrier gas. Of these 794 902 patterns, 150 200 were discarded due to instabilities in the electrospray, during which very large droplets were produced. The other 644 702 patterns were first converted to photons using previously described procedures,[4] and then classified into 50 classes using the *Dragonfly* software.[5] This classification is performed using the EMC algorithm,[6] where intensity models on the detector are determined which maximize the likelihood of generating the observed diffraction patterns using a Poisson noise model. The process was repeated from a random initial guess 5 times, yielding very similar results.

The results of this classification are shown in Figure S1 and Figure S2, in the classes labeled S1-S49. A second round of classification was performed with all patterns belonging to class averages which deviated from dense spherical particles, the results of which are marked as A1-A11 in the same figures.

Size determination SP-SAXS sphere classes:

The sphere classes were modeled using a custom Python script. The spherical form factor $f(q, r)$ is given by

$$f(q,r) \;=\; \frac{\sin(qr) - qr\cos(qr)}{(qr)^3}, \tag{S1}$$

where q is the magnitude of the scattering vector and $r = D/2$ the particle radius, and the intensity of monodisperse spherical particles would be

$$I_{\text{mono}}(q) \;\propto\; f^2(q,r). \tag{S2}$$

where the intensity is measured in photons per detector pixel. The proportionality constant depends on the incident X-ray fluence on the particle, average atomic scattering factor of the material and other geometrical factors like detector position, pixel size, X-ray polarization etc.[7] In the current experiment, the incident fluence varies randomly depending on which part of the focussed XFEL beam intercepts the particle, making it impossible to independently evaluate the scattering factor from the measured signal strength.

To account for size polydispersity within a class, we assume a Gaussian probability density function $p(r_0, \sigma)$ of the particle radii,

$$p(r, r_0, \sigma) \;=\; \frac{1}{\sqrt{2\pi}\,\sigma} \exp\left[-\frac{(r-r_0)^2}{2\sigma^2}\right], \tag{S3}$$

where r_0 is the mean radius and σ is the standard deviation. The scattering intensity of a polydisperse ensemble, $I_{\text{poly}}(q)$, is then calculated by summing the form-factor contributions of N discrete radii in a $\pm 3\sigma$-range weighted by their probability:

$$I_{\text{poly}}(q) \;\propto\; f^2_{\text{poly}}(q, r_0, \sigma) \;=\; \sum_{n=1}^{N} p(r_n, r_0, \sigma) \cdot f^2(q, r_n). \tag{S4}$$

To reduce the influence of the high-intensity low-q region during fitting, we minimize the residual χ^2 using relative intensity weights via

$$\chi^2 \;=\; \sum_i \left[\frac{I_i - I_{\text{model}}(q_i)}{I_i}\right]^2. \tag{S5}$$

The parameters r_0, σ, and a scale factor were refined by minimizing χ^2 using the Levenberg–Marquardt algorithm implemented in SciPy's optimize.least_squares function.[8]

Size determination SP-SAXS assembly classes:

The particle sizes of the assembly classes were estimated by assuming a uniform density for the spherical assemblies, following the approach of Grote et al.[9] The SP-SAXS profiles do not extend into the Guinier regime, which prevents reliable form-factor fitting. Instead, the assembly sizes were obtained directly from the position of the first intensity oscillation in the scattering profile. Specifically, we determine the size from either the maximum or the subsequent local minimum of the first intensity bump, as illustrated in Figure S3.

The zero of the first derivative of equation S1 and S2 gives the particle diameter of the first intensity oscillation maximum $q_{1,\text{max}}$ and the subsequent minimum $q_{2,\text{min}}$ via:

$$D_{1,\text{max}} = \frac{11.54}{q_{1,\text{max}}}, \qquad D_{2,\text{min}} = \frac{15.45}{q_{2,\text{min}}}. \tag{S6}$$

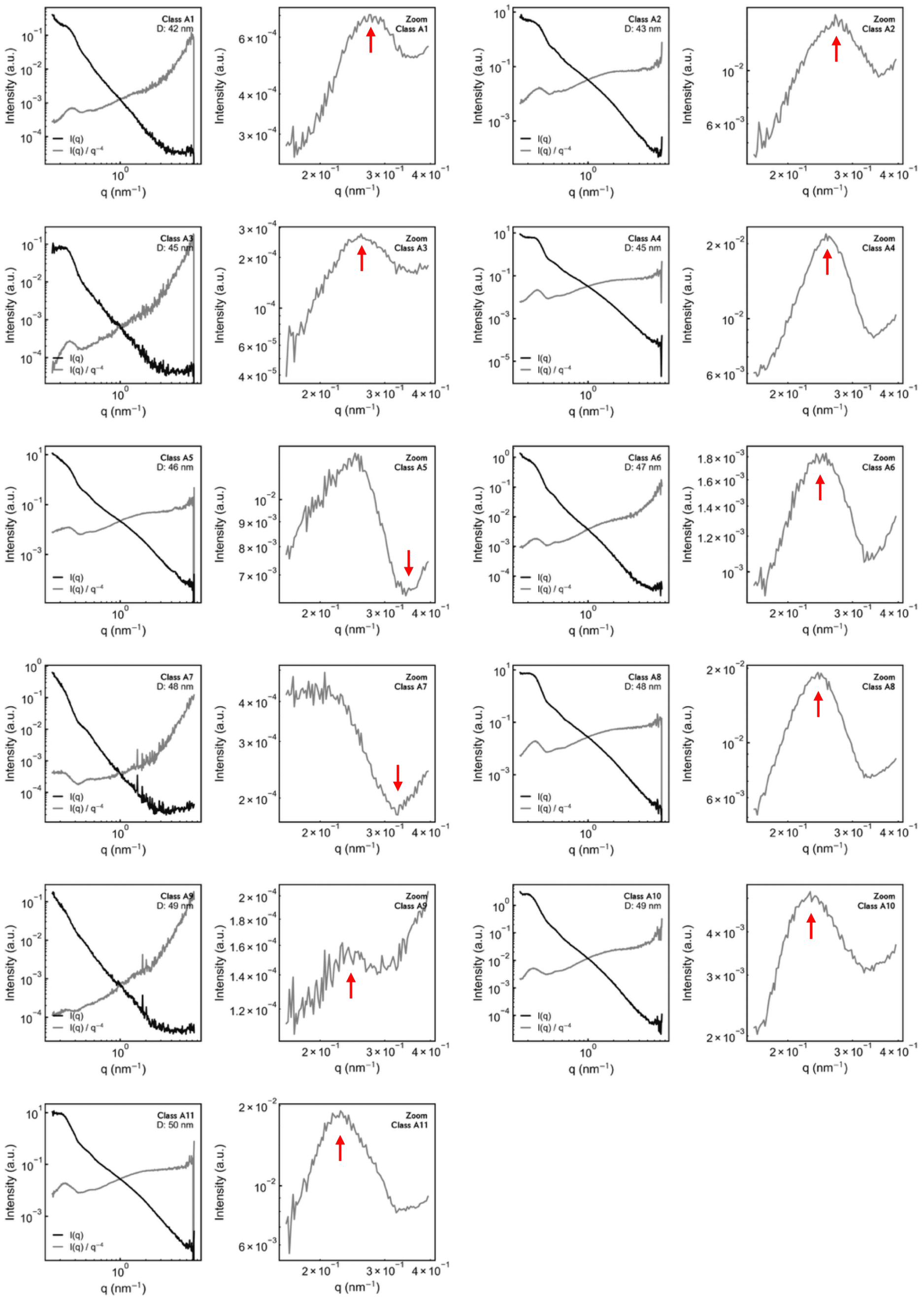

Figure S3: Radial integration of SP-SAXS assembly classes. The upwards facing red arrow indicates the local maximum, $q_{1,\mathrm{max}}$, and the downwards facing red arrow indicates subsequent minimum, $q_{2,\mathrm{min}}$, of the first intensity bump, which was used to determine the size of the assembly. To better see the intensity bump the data is divided by q^{-4}.

Synthesis:

All chemicals were purchased from commercial sources and used without further purification: $Co(acac)_3$ (Sigma-Aldrich, 99.99%), $Co(acac)_2$ (Acros, 99.9%), benzyl alcohol (Sigma-

Aldrich, >99%), and ethanol (VWR, absolute grade).

The synthesis is performed as described by Grote et al..[9] $Co(acac)_3$ (179.1 mg, 0.5 mmol) is added to 5 mL of benzyl alcohol and stirred for 10 min at room temperature. 0.8 mL of the reaction solution ist then transferred to the reaction container of the reactor, which is described in detail by Grote et al.. After assembling the reaction container in the reactor, it is first heated to 60 °C with a heating rate of 1 °C/s for 5 min, and then heated to 160 °C with the same heating rate. The time of the beginning of the reaction (t_0) is defined at the point where the heating of the reaction solution from 60 °C to 160 °C starts. All mentions of the reaction times are relative to t_0. For the SP-SAXS measurements the reaction was stopped after 20, 30, and 40 min, and the reactor was cooled with a cold metal block. Figures S4, S5, and S6 show conventional SAXS, PDF, and TEM data, respectively, of the 20, 30, and 40 min samples.

The samples for the reference EM measurements of $Co(acac)_2$ precipitates shown in Figure S7c,d were prepared by stirring $Co(acac)_2$ (55.4 mg, 0.2 mmol) in 2 mL benzyl alcohol or ethanol for 30 min at room temperature.

Sample preparation:

The SP-SAXS samples were prepared by centrifuging the quenched reaction solution in ethanol for 5 min at 3500 rpm, discarding the supernatant, and collecting the residue. The residue was then weighed and redispersed in a 10 mmol ammonium acetate ethanol solution to obtain a concentration of 0.075 mg/mL.

Conventional SAXS samples were prepared either by directly filling the reaction solution in a capillary or by centrifuging at 3500 rpm for 5 min in ethanol, redispersing the residue in ethanol and filling the capillary with the dispersion.

PDF samples were measured from dry powder after centrifuging at 3500 rpm for 5 min in ethanol.

EM samples were prepared by depositing one drop of the sample solution on a TEM grid

and washing the grid with a few drops of ethanol.

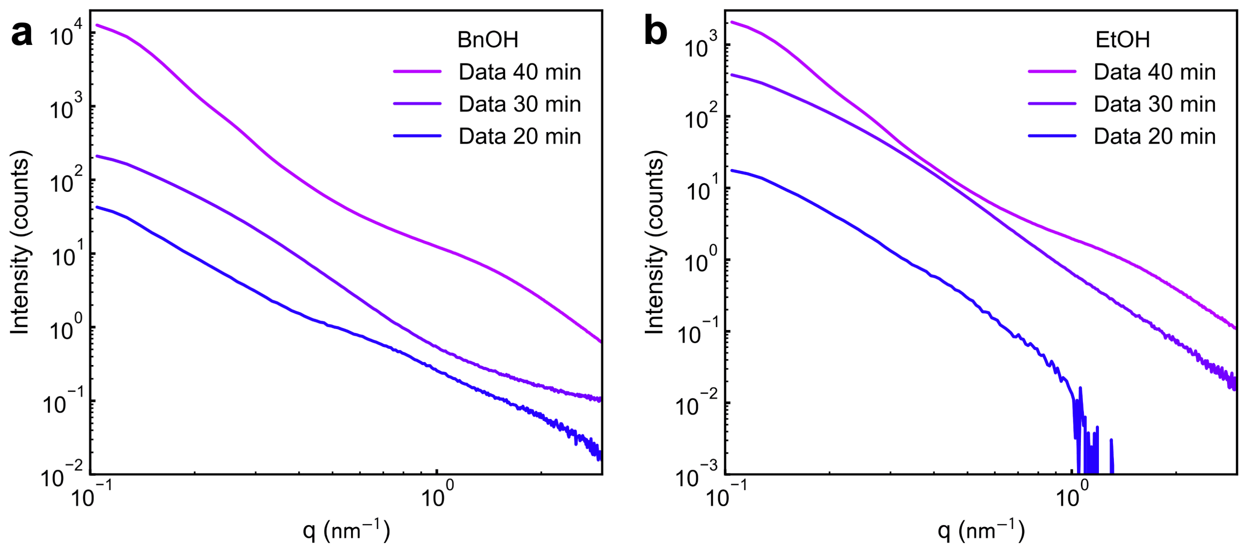

Figure S4: Conventional SAXS data of (a) reaction solutions in benzyl alcohol (BnOH) after 20, 30, and 40 min reaction time and (b) the reaction solutions redispersed in ethanol (EtOH) after centrifuging at 3500 rpm for 5 min. Both the BnOH and EtOH data show similar scattering profiles at the respective time points. Both data sets were measured at the lower part of capillary, which showed sample precipitation, making a comparison of the conventional SAXS to the summed SP-SAXS data, which is measured from very diluted particles, difficult.

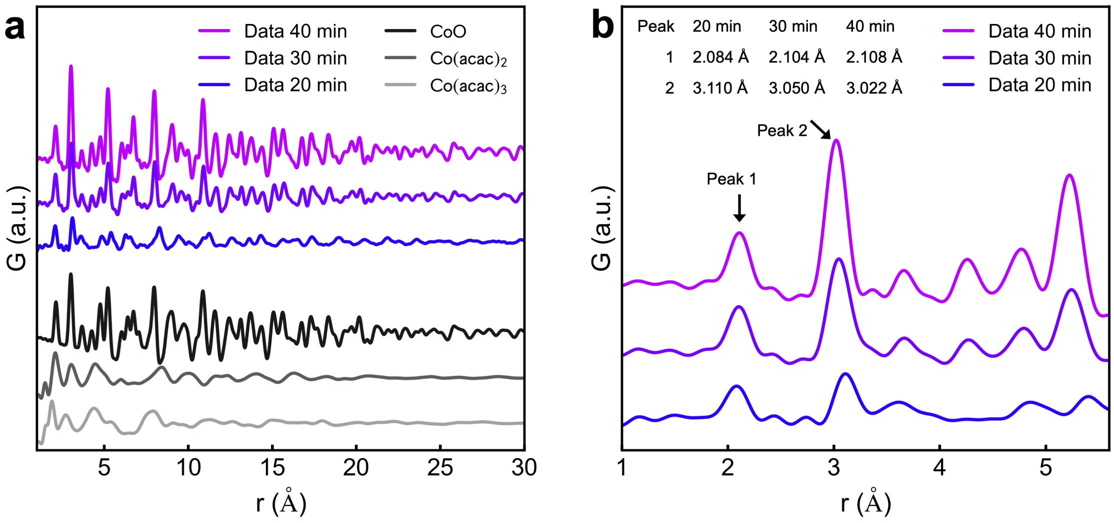

Figure S5: (a) Pair distribution function (PDF) of total X-ray scattering data of 20, 30, and 40 min samples compared to PDF simulations of rock-salt CoO, $Co(acac)_2$,[10] and $Co(acac)_3$[11] structures. The 30 and 40 min PDF closely matches the CoO simulation, while the 20 min sample shows features of both CoO and $Co(acac)_2$, which confirms the proposed phase transition of $Co(acac)_2$ to CoO. (b) Zoom of the experimental PDFs. The inset lists the peak position of the first and second peak. A detailed PDF analysis is beyond the scope of this paper, but available in the literature.[9]

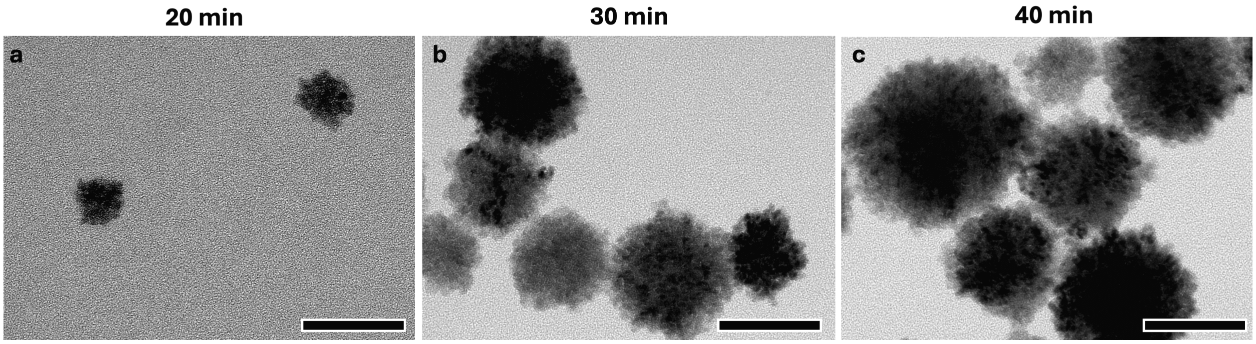

Figure S6: TEM images of CoO nanocrystal assemblies of reaction aliquots of (a) 20 min, (b) 30 min, and (c) 40 min. Scale bars 50 nm.

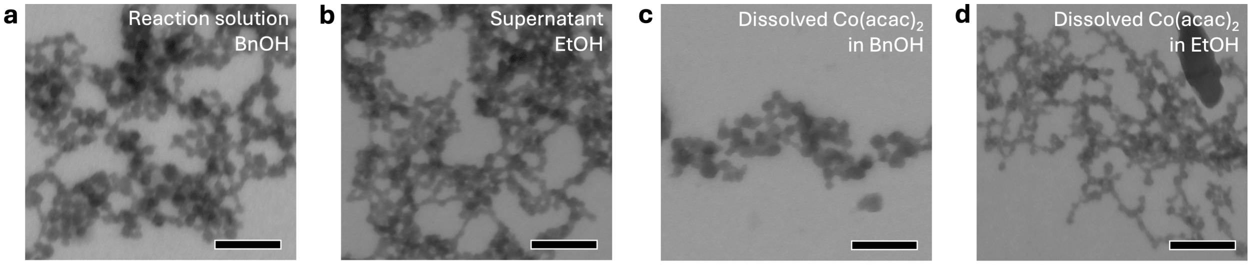

Figure S7: STEM images of spherical aggregates from solutions in benzyl alcohol (BnOH) or ethanol (EtOH). (a) Quenched reaction solution of the reaction of $Co(acac)_3$ in BnOH after 20 min reaction time. (b) Supernatant of washed reaction solution of (a) after centrifugation for 5 min at 3500 rpm in EtOH. Control experiments: commercial $Co(acac)_2$ dissolved in (c) BnOH and (d) EtOH for 30 min. Scale bars: 100 nm.

Origin of the apparent shift scattering minimum:

The class-averaged SAXS patterns show a broad minimum near $q = 2.5\,\mathrm{nm}^{-1}$, whose exact position varies between class averages. We examined whether this shift could reflect changes in the internal structure or composition of the amorphous intermediate particles. In principle, the position of this minimum depends on the relative contributions of the finite-size form factor and the higher-q scattering associated with short-range molecular or amorphous order. A shift in the minimum could therefore arise from changes in the relative volume fractions of different components, density modulations, or intermolecular correlations within the particles.

However, Fig. S8 shows how the position of the minimum is strongly correlated with the fitted width of the particle-size distribution contributing to each class average. Class averages containing a broader distribution of particle diameters show a systematically shifted and broadened minimum. This indicates that the apparent variation in q_{min} is dominated by averaging over particles with different sizes, rather than by a uniquely resolvable structural difference between the corresponding particle classes. We therefore do not assign a direct physical interpretation to the absolute value of q_{min} in individual class averages. Future measurements combining single-particle SAXS with simultaneous WAXS may allow such changes to be separated from size-distribution effects by directly probing the evolution of short-range order.

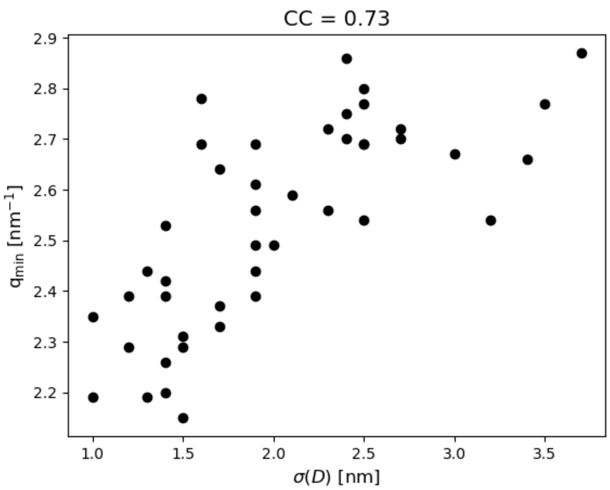

Figure S8: Correlation between fitted position of minima in SAXS curve in Fig. 2 ($q_{\min}$) against the size distribution of the particles in the class average.

Reproducibility of the synthesis:

The reaction kinetics observed in this study appear slightly slower than those reported by Grote et al.[9] based on the comparison of SAXS, EM, and PDF data. This difference is related to slight variations in the reactor inlet design, as discussed elsewhere.[12] The overall trends are similar.

Furthermore, we occasionally observe crumpled sheet- or rose-like particles with sizes of approximately 0.5–1.0 μm, as shown in Figure S9. These particles are beyond the detection limit of our SP-SAXS set-up and appear more frequently at 20 min, while they are rarely seen at 30 or 40 min. Similar rose-like structures were also reported by Grote et al.[9] at reaction times earlier than 20 min. Given their disappearance as the reaction progresses, we attribute these particles to side reactions that do not affect the formation of CoO nanocrystal assemblies.

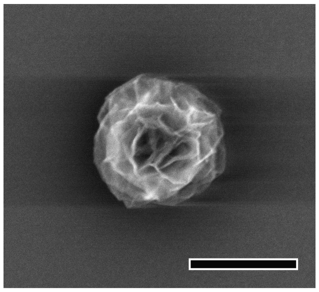

Figure S9: SEM image of a rose-like particle from a 20 min reaction aliquot. Scale bar: 500 nm.

Electron microscopy (EM):

Scanning electron microscopy (SEM) and scanning transmission electron microscopy (STEM) images were collected using a Regulus 8220 (Hitachi High Technologies Corp.) with an acceleration voltage of 30 keV. Transmission electron microscopy (TEM) images were collected using a JEM 1011 (JEOL Ltd.) with an acceleration voltage of 100 keV.

Conventional SAXS:

SAXS data was acquired at beamline P62 of PETRA III at Deutsches Elektronen–Synchrotron DESY, Hamburg, Germany. The particle dispersions were filled in a 1 mm diameter borosilicate capillary and diffraction images were recorded for 30 s at an X-ray energy of 24.30 keV (λ = 0.5102 Å) using a two-dimensional X-ray detector (EIGER2 X 9M, Dectris Ltd.) with 3108×3262 pixels and a pixel size of 75×75 µm^2 and a sample-to-detector distance of 4.946 m, obtained from a calibration with a silver behenate standard packed into a capillary. The diffraction images were integrated using PyFAI.[13]

Total X-ray scattering (TS) and pair distribution function (PDF) analysis:

TS data was acquired at beamline P21.1[14] of PETRA III at Deutsches Elektronen–Synchrotron DESY, Hamburg, Germany. The powder sample was packed in a 1 mm diameter borosilicate capillary and diffraction images were recorded for 60 s at an X-ray energy of 101.39 keV (λ = 0.1222 Å) using a two-dimensional X-ray detector (PerkinElmer XRD1621, Varex Imaging Corp.) with 2048×2048 pixels and a pixel size of 200×200 µm^2 and a sample-to-detector distance of 0.301 m, obtained from a calibration with a LaB_6 powder standard packed into a cap-

illary. The diffraction images were integrated using PyFAI.[13] The experimental PDFs were calculated using PDFgetX3[15] with values $q_{max,inst}$ =25.0 Å, q_{max} =22.5 $Å^{-1}$, q_{min} =1.0 $Å^{-1}$, and r_{poly} = 0.9. The PDF simulations were calculated using DiffPy-CMI[16] with the same values as the experimental PDF calculations.

References

(1) Mancuso, A. P.; Aquila, A.; Batchelor, L.; Bean, R. J.; Bielecki, J.; Borchers, G.; Doerner, K.; Giewekemeyer, K.; Graceffa, R.; Kelsey, O. D.; Kim, Y.; Kirkwood, H. J.; Legrand, A.; Letrun, R.; Manning, B.; Lopez Morillo, L.; Messerschmidt, M.; Mills, G.; Raabe, S.; Reimers, N.; Round, A.; Sato, T.; Schulz, J.; Signe Takem, C.; Sikorski, M.; Stern, S.; Thute, P.; Vagovič, P.; Weinhausen, B.; Tschentscher, T. The single particles, clusters and biomolecules and serial femtosecond crystallography instrument of the European XFEL: initial installation. *Synchrotron Radiation* **2019**, *26*, 660–676.

(2) Bielecki, J.; Hantke, M. F.; Daurer, B. J.; Reddy, H. K. N.; Hasse, D.; Larsson, D. S. D.; Gunn, L. H.; Svenda, M.; Munke, A.; Sellberg, J. A.; Flueckiger, L.; Pietrini, A.; Nettelblad, C.; Lundholm, I.; Carlsson, G.; Okamoto, K.; Timneanu, N.; Westphal, D.; Kulyk, O.; Higashiura, A.; van der Schot, G.; Loh, N.-T. D.; Wysong, T. E.; Bostedt, C.; Gorkhover, T.; Iwan, B.; Seibert, M. M.; Osipov, T.; Walter, P.; Hart, P.; Bucher, M.; Ulmer, A.; Ray, D.; Carini, G.; Ferguson, K. R.; Andersson, I.; Andreasson, J.; Hajdu, J.; Maia, F. R. N. C. Electrospray sample injection for single-particle imaging with x-ray lasers. *Science Advances* **2019**, *5*, eaav8801.

(3) Allahgholi, A.; Becker, J.; Delfs, A.; Dinapoli, R.; Göttlicher, P.; Graafsma, H.; Greiffenberg, D.; Hirsemann, H.; Jack, S.; Klyuev, A.; Krüger, H.; Kuhn, M.; Laurus, T.; Marras, A.; Mezza, D.; Mozzanica, A.; Poehlsen, J.; Shefer Shalev, O.; Sheviakov, I.; Schmitt, B.; Schwandt, J.; Shi, X.; Smoljanin, S.; Trunk, U.; Zhang, J.; Zimmer, M. Megapixels@ Megahertz–The AGIPD high-speed cameras for the European XFEL. *Nu-*

clear Instruments and Methods in Physics Research Section A: Accelerators, Spectrometers, Detectors and Associated Equipment **2019**, *942*, 162324.

(4) Ayyer, K.; Xavier, P. L.; Bielecki, J.; Shen, Z.; Daurer, B. J.; Samanta, A. K.; Awel, S.; Bean, R.; Barty, A.; Bergemann, M.; Ekeberg, T.; Estillore, A. D.; Fangohr, H.; Giewekemeyer, K.; Hunter, M. S.; Karnevskiy, M.; Kirian, R. A.; Kirkwood, H.; Kim, Y.; Koliyadu, J.; Lange, H.; Letrun, R.; Lübke, J.; Michelat, T.; Morgan, A. J.; Roth, N.; Sato, T.; Sikorski, M.; Schulz, F.; Spence, J. C. H.; Vagovic, P.; Wollweber, T.; Worbs, L.; Yefanov, O.; Zhuang, Y.; Maia, F. R. N. C.; Horke, D. A.; Küpper, J.; Loh, N. D.; Mancuso, A. P.; Chapman, H. N. 3D diffractive imaging of nanoparticle ensembles using an x-ray laser. *Optica* **2021**, *8*, 15.

(5) Ayyer, K.; Lan, T.-Y.; Elser, V.; Loh, N. D. *Dragonfly* : an implementation of the expand–maximize–compress algorithm for single-particle imaging. *Journal of Applied Crystallography* **2016**, *49*, 1320–1335.

(6) Loh, N.-T. D.; Elser, V. Reconstruction algorithm for single-particle diffraction imaging experiments. *Physical Review E—Statistical, Nonlinear, and Soft Matter Physics* **2009**, *80*, 026705.

(7) Daurer, B. J.; Okamoto, K.; Bielecki, J.; Maia, F. R. N. C.; Mühlig, K.; Seibert, M. M.; Hantke, M. F.; Nettelblad, C.; Benner, W. H.; Svenda, M.; Tîmneanu, N.; Ekeberg, T.; Loh, N. D.; Pietrini, A.; Zani, A.; Rath, A. D.; Westphal, D.; Kirian, R. A.; Awel, S.; Wiedorn, M. O.; van der Schot, G.; Carlsson, G. H.; Hasse, D.; Sellberg, J. A.; Barty, A.; Andreasson, J.; Boutet, S.; Williams, G.; Koglin, J.; Andersson, I.; Hajdu, J.; Larsson, D. S. D. Experimental strategies for imaging bioparticles with femtosecond hard X-ray pulses. *IUCrJ* **2017**, *4*, 251–262.

(8) Virtanen, P.; Gommers, R.; Oliphant, T. E.; Haberland, M.; Reddy, T.; Cournapeau, D.; Burovski, E.; Peterson, P.; Weckesser, W.; Bright, J.; van der Walt, S. J.;

Brett, M.; Wilson, J.; Millman, K. J.; Mayorov, N.; Nelson, A. R. J.; Jones, E.; Kern, R.; Larson, E.; Carey, C. J.; Polat, I.; Feng, Y.; Moore, E. W.; VanderPlas, J.; Laxalde, D.; Perktold, J.; Cimrman, R.; Henriksen, I.; Quintero, E. A.; Harris, C. R.; Archibald, A. M.; Ribeiro, A. H.; Pedregosa, F.; van Mulbregt, P. SciPy 1.0: fundamental algorithms for scientific computing in Python. *Nature Methods* **2020**, *17*, 261–272.

(9) Grote, L.; Zito, C. A.; Frank, K.; Dippel, A.-C.; Reisbeck, P.; Pitala, K.; Kvashnina, K. O.; Bauters, S.; Detlefs, B.; Ivashko, O.; Pandit, P.; Rebber, M.; Harouna-Mayer, S. Y.; Nickel, B.; Koziej, D. X-ray studies bridge the molecular and macro length scales during the emergence of CoO assemblies. *Nature Communications* **2021**, *12*, 4429.

(10) Vreshch, V. D.; Yang, J.-H.; Zhang, H.; Filatov, A. S.; Dikarev, E. V. Monomeric Square-Planar Cobalt(II) Acetylacetonate: Mystery or Mistake? *Inorganic Chemistry* **2010**, *49*, 8430–8434.

(11) Chrzanowski, L. S. v.; Lutz, M.; Spek, A. L. α-Tris(2,4-pentanedionato-$\kappa^2 O,O'$)cobalt(III) at 240, 210, 180, 150 and 110K. *Acta Crystallographica Section C: Crystal Structure Communications* **2007**, *63*, m283–m288.

(12) Harouna-Mayer, S. Y.; Gumus Akcaalan, M.; Kopula Kesavan, J.; Groene, T. R. L.; Klemeyer, L.; Hussak, S.-A.; Grote, L.; Derelli, D.; Caddeo, F.; Zito, C.; Stützle, P.; Speer, D.; Dippel, A.-C.; Detlefs, B.; Appiarius, Y.; Jacobi Von Wangelin, A.; Koziej, D. Modular reactor for *in situ* X-ray scattering, spectroscopy and ATR-IR studies of solvothermal nanoparticle synthesis. **2026**, *33*, 142–152.

(13) Kieffer, J.; Wright, J. P. PyFAI: a Python library for high performance azimuthal integration on GPU. *Powder Diffraction* **2013**, *28*, S339–S350.

(14) v. Zimmermann, M.; Ivashko, O.; Igoa Saldaña, F.; Liu, J.; Glaevecke, P.; Gutowski, O.;

Nowak, R.; Köhler, K.; Winkler, B.; Schöps, A.; Schulte-Schrepping, H.; Dippel, A.-C. P21.1 at PETRA III – a high-energy X-ray diffraction beamline for physics and chemistry. *Journal of Synchrotron Radiation* **2025**, *32*, 802–814.

(15) Juhás, P.; Davis, T.; Farrow, C.; Billinge, S. *PDFgetX3* : a rapid and highly automatable program for processing powder diffraction data into total scattering pair distribution functions. *Journal of Applied Crystallography* **2013**, *46*, 560–566.

(16) Juhás, P.; Farrow, C.; Yang, X.; Knox, K.; Billinge, S. Complex modeling: a strategy and software program for combining multiple information sources to solve ill posed structure and nanostructure inverse problems. *Acta Crystallographica Section A: Foundations and Advances* **2015**, *71*, 562–568.
