# Supplementary material for: Single-Particle X‑ray Scattering Reveals a High Local Supersaturation of Precursors as the Origin of CoO Assembly Formation
Source: J Phys Chem Lett. 2026 Jun 1;17(23):6418–24. doi: 10.1021/acs.jpclett.6c00191 (PMC13267086; doi:10.1021/acs.jpclett.6c00191)
Supplement: Supplementary file 2 [file jz6c00191_si_002.pdf]

jz-2026-00191v.R1

Name: Peer Review Information for "Single-Particle X-ray Scattering Reveals a High Local Supersaturation of Precursors as the Origin of CoO Assembly Formation"

First Round of Reviewer Comments

Reviewer: 1

Comments to the Author

The authors have reported Single-particle small-angle X-ray scattering (SP-SAXS) results perhaps for the first time measured at the European X-ray free electron lasers (XFEL) facility. Methods developed to analyze the collected data is quite good and results obtained from the measurement is very nice. However there are some issues regarding interpretation of the results as I have outlined below. The manuscript may be accepted after the authors incorporate the changes indicated below.

1. Although the studied CoO nanocrystal assemblies are considered as cavernous, it is important to present evolution of scattering amplitude as a function of time. This will require replacement of proportionality by a time-dependent factor in equation (2) and corresponding changes in equation (4). The evolution of this time-dependent factor should be shown.

2. The broad minima around q = 2.5 nm(-1) in Fig. 2(d) is shifting to a higher value in Fig. 2(e) - it should be marked clearly and discussed in the revised manuscript.

Reviewer: 2

Comments to the Author

The manuscript presents an important work on application XFEL based SAXS measurements which they claim as single particle measurement. Using the study they show a detailed mechanistic understanding of growth of CoO nanocrystal assembly especially where they are able to capture the intermediate states of formation. The claim of the manuscript is interesting where they present a new technique for detailed growth dynamics study in nanoparticles, nanoclusters and assemblies which is inaccessible to conventional techniques. However the manuscript needs better clarity before further consideration.

Though the technique employed is a state of art method however the manuscript needs clear justifications especially on the following aspects for establishing the claim of SPSAXS and the potential of XFEL in similar studies compared to conventional synchrotron based SAXS measurements:

1. It is unclear how the measurement captures single particle and not averaged over multi particle as the beam spot is expected to be orders of magnitude larger than a single particle. In absence of clear justification of this it will be effectively an ensemble averaged measurement as performed using other conventional techniques as synchrotron based SAXS/WAXS.

2. Advantage of use of XFEL is unclear as the dynamics discussed is of the order of tens of minutes. Authors should discuss if the ultrafast probe time scales available in XFEL is of any particular use. Especially it will be important to present data on the fast dynamics in these studies.

Author's Response to Peer Review Comments:

Dear Editor,
Please find attached our response to reviewers below. Our response is in **blue**, while changes to the manuscript are quoted in red.

# Reviewer 1

The authors have reported Single-particle small-angle X-ray scattering (SP-SAXS) results perhaps for the first time measured at the European X-ray free electron lasers (XFEL) facility. Methods developed to analyze the collected data is quite good and results obtained from the

measurement is very nice. However there are some issues regarding interpretation of the results as I have outlined below. The manuscript may be accepted after the authors incorporate the changes indicated below.
We thank the reviewer for their favorable comments. Please find our responses to their specific concerns below.

1. Although the studied CoO nanocrystal assemblies are considered as cavernous, it is important to present evolution of scattering amplitude as a function of time. This will require replacement of proportionality by a time-dependent factor in equation (2) and corresponding changes in equation (4). The evolution of this time-dependent factor should be shown.
The reviewer is correct that an absolute measure of the average electron density in the assemblies would be good evidence of their cavernous nature. Unfortunately, in a single-particle imaging configuration, this is difficult since the incident fluence on any given particle is unknown. The particles flow past the X-ray focus and while some of them are intercepted near the center of the focus, others are intercepted in the tails. The prefactor in equation 2 contains both the scattering cross-section as well as the incident fluence, which are difficult to detangle.

We therefore rely on the dependence of the SAXS signal versus *q* to identify density modulations within the assembly. While these density variations are not conclusively voids (rather than density hotspots), the evidence from TEM and from the prior Grote et al. synchrotron studies suggest our model. This is also consistent with the formation of CoO crystallites which have a higher packing fraction than amorphous $Co(acac)_2$ aggregates.

We added the following clarification to Eqn. (2):
…where the intensity is measured in photons per detector pixel. The proportionality constant depends on the incident X-ray fluence on the particle, average atomic scattering factor of the material and other geometrical factors like detector position, pixel size, X-ray polarization etc. (Daurer, *IUCrJ* 2017). In the current experiment, the incident fluence varies randomly depending on which part of the focussed XFEL beam intercepts the particle, making it impossible to independently evaluate the scattering factor from the measured signal strength.

2. The broad minima around q = 2.5 nm(-1) in Fig. 2(d) is shifting to a higher value in Fig. 2(e) - it should be marked clearly and discussed in the revised manuscript.

We must concede that we actually did not notice the fact that the minima is at a different *q* for different class averages. We thank the reviewer for pointing this out and leading us to analyze this feature further.

In general, the position of the minima is related to the relative strength of the sphere form factor and the amorphous pair distribution function signal which starts to appear at higher *q*.

Variation in this $q_{min}$ could indicate different volume fractions of different components within the amorphous droplet, or other variations in intermolecular interactions.

Unfortunately, after more investigation, it appears that the position of the minima is strongly correlated with the size variation in each class average. Since each single shot pattern does not have enough signal at that *q* to be visible over background, we average "similar" patterns to generate the class average patterns in Fig. 2d and 2e.

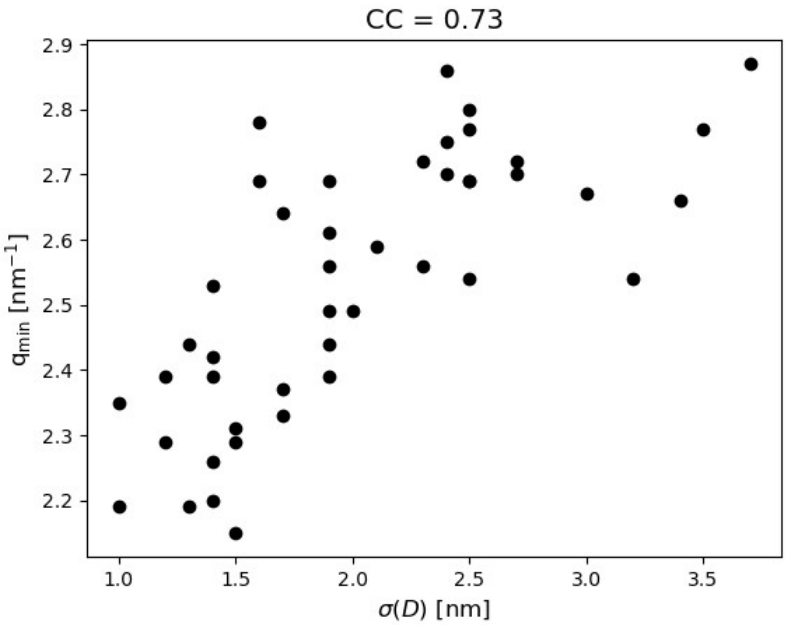


The scatter plot above shows the dependence of $q_{min}$ on the fitted standard deviation of diameters of the particle's contribution to a given class average. Thus, it appears that this is an artifact of the averaging process and does not appear to have a physical interpretation.

In future experiments, we hope to be able to make simultaneous WAXS measurements to see whether the position and strength of the high-*q* maxima depend on reaction time and particle size, providing a glimpse into short range interactions within these amorphous clusters.

We have added this information as a separate Supporting Information Section.

*Origin of the apparent shift scattering minimum*:

The class-averaged SAXS patterns show a broad minimum near q = 2.5 $nm^{-1}$, whose exact position varies between class averages. We examined whether this shift could reflect changes in the internal structure or composition of the amorphous intermediate particles. In principle, the position of this minimum depends on the relative contributions of the finite-size form factor and the higher-*q* scattering associated with short-range molecular or amorphous order. A shift in the minimum could therefore arise from changes in the relative volume fractions of different components, density modulations, or intermolecular correlations within the particles.

However, Fig. S8 shows how the position of the minimum is strongly correlated with the fitted width of the particle-size distribution contributing to each class average. Class averages containing a broader distribution of particle diameters show a systematically shifted and broadened minimum. This indicates that the apparent variation in $q_{min}$ is dominated by averaging over particles with different sizes, rather than by a uniquely resolvable structural difference between the corresponding particle classes. We therefore do not assign a direct physical interpretation to the absolute value of $q_{min}$ in individual class averages. Future measurements combining single-particle SAXS with simultaneous WAXS may allow such changes to be separated from size-distribution effects by directly probing the evolution of short-range order.

# Reviewer 2

The manuscript presents an important work on application XFEL based SAXS measurements which they claim as single particle measurement. Using the study they show a detailed mechanistic understanding of growth of CoO nanocrystal assembly especially where they are able to capture the intermediate states of formation. The claim of the manuscript is interesting where they present a new technique for detailed growth dynamics study in nanoparticles, nanoclusters and assemblies which is inaccessible to conventional techniques. However the manuscript needs better clarity before further consideration.

Though the technique employed is a state of art method however the manuscript needs clear justifications especially on the following aspects for establishing the claim of SPSAXS and the potential of XFEL in similar studies compared to conventional synchrotron based SAXS measurements:

1. It is unclear how the measurement captures single particle and not averaged over multi particle as the beam spot is expected to be orders of magnitude larger than a single particle. In absence of clear justification of this it will be effectively an ensemble averaged measurement as performed using other conventional techniques as synchrotron based SAXS/WAXS.

We apologize for the lack of clarity in the manuscript about this aspect. While the beam spot (250-300 nm) is much larger than the particle size, the aerosolized particles are at a density that, on average, much less than one particle is in the beam for any pulse. Each detector frame measures the signal from one particle. This noisy signal is then classified and averaged to create the class averages shown in Fig. 2. We attempt to illustrate this process in Fig. 1, but now see how this is not so clear.

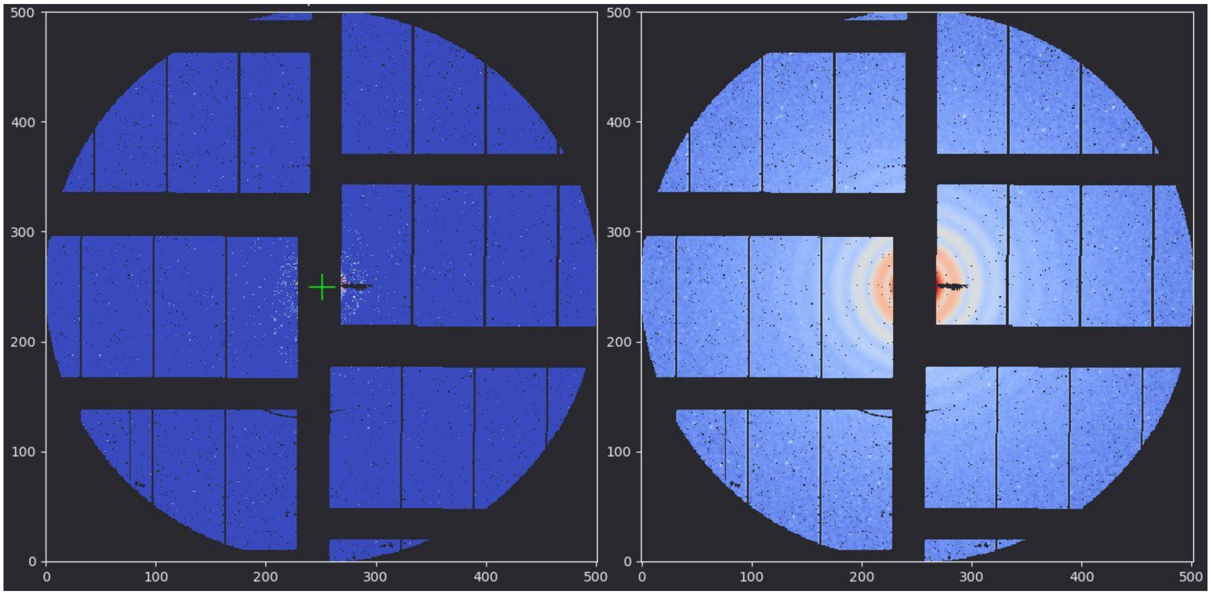


The left panel represents the inner region of a single detector frame. The light blue/white dots are single photons and the dark regions are panel and ASIC gaps and masked pixels. The right panel shows the corresponding predicted class average intensity on a logarithmic scale.

The downstream analysis is performed with the class averages after subtracting the “no-hit” background. We have made the following changes to manuscript to make this clear:

Although the focused XFEL beam, with a diameter of approximately 250–300 nm, is larger than the particles, most X-ray pulses therefore interact with no particle, while a small fraction produce a measurable single-particle scattering pattern. Since individual single-shot patterns contain only a few photons at high *q*, structurally similar patterns are classified and averaged for further analysis. In Figure 2, we show representative aligned average diffraction patterns of selected classes.

2. Advantage of use of XFEL is unclear as the dynamics discussed is of the order of tens of minutes. Authors should discuss if the ultrafast probe time scales available in XFEL is of any articular use. Especially it will be important to present data on the fast dynamics in these studies.

The reviewer is correct that the biggest perceived advantage of XFELs is their short pulse duration, enabling one to observe ultrafast structural dynamics. In other work, we have capitalized on this feature to study optically excited nanoparticles (Shen, et al *ACS Nano* 19, 32 (2025)).

However another distinguishing feature of XFELs is their extremely high peak intensity, with each XFEL pulse containing as many coherent photons as generated in 1 second at a 3rd generation synchrotron source. This enables one to look at single particles, one at a time,

rather than ensemble averaging. In this work, we have attempted to use this property of XFELs to observe a previously unobserved intermediate in a relatively slow synthetic process. The ultrashort nature of the pulses is still relevant in outrunning radiation damage effects, but not directly in a time-resolved experiment. We have commented on this aspect in the conclusions:

Although the reaction studied here evolves on minute time scales, the XFEL enables single-particle analysis from rare intermediates, avoiding averaging of details due to heterogeneity. The femtosecond pulse duration freezes the particle structure before radiation damage develops, while the high peak coherent flux provides sufficient signal from one particle at a time. The present experiment therefore illustrates a complementary use of XFELs in materials chemistry: not only for femtosecond dynamics, but also for measuring heterogeneous structural distributions in relatively slow, non-equilibrium synthesis processes.
